# On Modern Progress in Ophthalmic Medicine and Surgery

**Published:** 1894-10-06

**Authors:** Robert Brudenell Carter

**Affiliations:** Consulting Ophthalmic Surgeon to St. George's Hospital


### ON MODERN PROGRESS IN OPHTHALMIC MEDICINE AND SURGERY.

By ROBERT BRUDENELL CARTER, F.R.C.S., Consulting Ophthalmic Surgeon to St. George's Hospital.

LAMINAR CATARACT (*continued*).

THE lens being broken up and softened, as described in the last paper, the resulting flocculent mass should be removed from the eye before it begins to excite any serious amount of irritation. In a young adult, or in a child of ten years old or more, it will be sufficient to render the eye insensitive by cocaine; but for younger children, who might be rendered unsteady by fear alone, it is prudent to use a general anæsthetic. The lids being widely separated by a spring speculum, the surgeon fixes the eye by pinching up a broad fold of conjunctiva on the nasal side, near the cornea, and with the other hand carries a small iridectomy knife into the anterior chamber. The puncture for this purpose should be in the cornea itself, on its temporal side, at about one-third of its semi-diameter from the margin. The point of the knife should be directed towards the centre of the eyeball, and should be pushed on until the opening is large enough to admit the suction tube easily. If properly made the opening will be valvular, and will gape when slight pressure is exerted upon the side nearer to the corneal margin. In the act of withdrawal, the back of the knife should be pressed against this marginal lip, and a good deal of the softened lens matter may then be expected to escape through the aperture. If any remain behind, the suction tube must be gently introduced, with its flat side and opening forwards, and be carried somewhat deeply into the space formerly occupied by the lens, so as to have the debris between its opening and the cornea. Very gentle mouth suction will draw all that remains into the tube, and any fragment which seems disposed to linger in the eye may be followed with the tube and captured. Sometimes a fragment will block the tube and interfere with further suction, and in that case the tube should be withdrawn, blown clear, and gently reintroduced. When all the visible lens matter has been removed the operation is concluded, and the eye may be closed with isinglass plaster in the ordinary way. If the incision has been correctly placed, the iris will be removed from contact with it as soon as the aqueous humour is re-secreted, and there will be no probability of any adhesion of the iris to the cicatrix. The eye should be examined within twenty-four hours, and should be kept fully under the influence of atropine until all probability of inflammatory reaction has passed away. When recovery is in all other respects complete, the pupil will often be more or less obstructed by a film of capsule, which, if it fail to undergo retraction within a reasonable time, must be divided with needles in the ordinary way.

If the first part of the operation, the division of the lens, has been imperfectly accomplished, leaving a thick posterior layer, or any other large portion, which the needles have failed to touch, such a portion may so far escape the action of the aqueous humour as to be still transparent, and therefore invisible, when suction is performed. The removal of the softened portions will expose more completely those which remain, and the introduction of the tube may complete the breaking up of lens matter which had previously escaped. The result will be that, when the eye is opened for examination, the anterior chamber will present much the same appearance that it did prior to the suction, being still occupied with opaque and flocculent material, and, what is still worse, being still exposed to the risks arising from the inflammation which this material may set up. In such circumstances it will be necessary to repeat suction as soon as possible, and, in the meanwhile, to use atropine diligently, and to apply leeches to the temple if iritis should supervene. The sooner the eye can be relieved from the whole of the lens material, the sooner will it be placed beyond the reach of danger.

When first introduced into English practice by Mr. Teale, the practice of suction was somewhat eagerly welcomed, and it was soon extensively employed with only imperfect regard to conditions which were essential to success. The results were in several cases unfortunate, insomuch that, by many surgeons, and even, I have been told, in some institutions, the method has been abandoned. Many years ago, at St. George's Hospital, I operated upon a boy in whom the suction was followed by septic inflammation of a destructive character. The hospital was about to be closed for alterations, and the patients were transferred to another, at which the eye in question was removed by the surgeon who took charge of the patient. It was the first unsuccessful case which I had seen, and I have not seen a second, although I have since performed the operation very frequently. I have no doubt that the septic inflammation was due to the conveyance into the eye, by means of the suction tube itself, of some albuminous matter in a state of change, the residue of some former operation. The silver portion of the suction tube is cemented to the glass portion by shellac, which is at once softened either by heat or by alcohol, so that the instrument can neither be put into hot water or into spirit without separation of its parts, and consequent disablement until it has been repaired. It can only be washed in cold water, which may leave fragments of lens matter in either the silver or the glass portion, and these fragments, when dry and easily detachable, may as easily be conveyed into the eye. Since I became aware of this source of danger, it has been my practice to soak the entire suction tube in an antiseptic solution for some hours before using it. If I am going to operate in the morning, I put the tube to soak over night. At one time I used diluted Condy's fluid, which is, I believe, perfectly effectual, but which tarnishes the silver and spoils the appearance of the instrument. I now use a 15 per cent. solution of Barff's boro-glyceride, and I have no accidents to deplore. In dealing with both eyes, my ordinary practice is to break up one lens, and to break up the second when I suck out the first. In this way the double operation for both eyes may be brought within the compass of three weeks; although, if one or both of the pupils should be obstructed by capsules, it is usually prudent to defer the use of the needles for at least another month, and often still longer.

When the subjects of laminar cataract have been suffered to reach middle age without operation, and it is then required, it will usually be prudent to perform ordinary extraction, or else to make a small upward iridectomy about a month or six weeks prior to discission and suction.

